# TLS Protocol Analysis Using IoTST—An IoT Benchmark Based on Scheduler Traces

**DOI:** 10.3390/s23052538

**Published:** 2023-02-24

**Authors:** Rafael Salles, Ricardo Farias

**Affiliations:** Systems Engineering and Computer Science Program (PESC/COPPE/UFRJ), Federal University of Rio de Janeiro, Rio de Janeiro 21941-972, Brazil

**Keywords:** IoT benchmarking, TLS 1.3, ECC, ECDSA, RSA, ECDHE, ESP32

## Abstract

The Internet of Things (IoT) envisions billions of everyday objects sharing information. As new devices, applications and communication protocols are proposed for the IoT context, their evaluation, comparison, tuning and optimization become crucial and raise the need for a proper benchmark. While edge computing aims to provide network efficiency by distributed computing, this article moves towards sensor nodes in order to explore efficiency in the local processing performed by IoT devices. We present IoTST, a benchmark based on per-processor synchronized stack traces with the isolation and precise determination of the introduced overhead. It produces comparable detailed results and assists in determining the configuration that has the best processing operating point so that energy efficiency can also be considered. On benchmarking applications which involve network communication, the results can be influenced by the constant changes that occur in the state of the network. In order to circumvent such problems, different considerations or assumptions were used in the generalization experiments and the comparison to similar studies. To present IoTST usage on a real problem, we implemented it on a commercial off the-shelf (COTS) device and benchmarked a communication protocol, producing comparable results that are unaffected by the current network state. We evaluated different Transport-Layer Security (TLS) 1.3 handshake cipher suites at different frequencies and with various numbers of cores. Among other results, we could determine that the selection of a specific suite (Curve25519 and RSA) can improve the computation latency by up to four times over the worst suite candidate (P-256 and ECDSA), while both providing the same security level (128 bits).

## 1. Introduction

One of the main requirements of the IoT device is to communicate through a wireless network while being low cost, energy efficient and having a small form factor [1].

Low-cost COTS devices are widely used in rapid-prototyping IoT environments [2,3]. Typically, the manufacturer provides both the hardware and the minimal related software, including proprietary code with no source (i.e., a compiled driver library). Such devices are treated as black boxes, and not much thought is given to performance [4,5]. The use of black-box devices without proper evaluations can have serious implications [5,6]. One challenge in this context is understanding the relative performances of different hardware/software configurations.

Performance benchmarking is the process of inducing stress on a system while observing its responses. Benchmarks can be classified as synthetic or application (“real world”) benchmarks [7]. Typically, synthetic or application-driven workloads are executed on a system while measuring quality characteristics, such as I/O throughput, end-to-end communication or computation latency [8]. A synthetic benchmark’s intent is to measure features of a system, processor or compiler. Some of these benchmarks, designed for supercomputers in the 1970s or 1980s, are still used. Two synthetic benchmark examples, ported for IoT devices, are the Linpack [9], which measures the floating-point rate of execution, and the Dhrystone [10], which benchmark the devices based on solving of a dense system of linear equations. Synthetic benchmarks are useful in debugging specific features, but they cannot be easily related to how those features will perform in an application [7]. Application benchmarks use system- or user-level software code drawn from real algorithms or full applications. They are more common in system-level benchmarking and usually have large code and data storage requirements. Typically, a timer measures each repetition, encompassing all the underlying processing.

A typical IoT application can be broken down into four main blocks: those for actuating/sensing, for data handling, for system management tasks and for networking (or data communication) [11,12]. In regard to the last block, the environment in which wireless networks are immersed changes constantly [13]. Any object movement can act as a barrier to microwave propagation. Another cause of such changes is the interference caused by neighboring equipment, such as other Wi-Fi devices or microwave ovens. These constant changes can result in packet drops, re-transmissions, link instability and inconsistent protocol behavior [14], which can compromise the results of an IoT benchmark.

In order to be considered complete, any benchmark must define [15]: a workload, a metric of comparison and rules for running the workload. As different IoT communication protocols and algorithms are being proposed, each study uses its own methodology under specific or not-mentioned wireless conditions. In most cases, comparison of results is not possible.

This paper proposes IoTST, an IoT application benchmark based on the collection and classification of scheduler traces. Using the IoTST methodology, we analyze a communication subsystem on a device so that the traces collected can identify when the system waits for network-data transmission. As a result, an experimental analysis using IoTST can isolate, and optionally discard, the processing that depends on the current network state, allowing the creation of a network-invariant benchmark.

The main contributions of this paper are:

- A review of benchmark studies that evaluate IoT applications or protocols for performing data communication;
- The development of a network-invariant benchmarking methodology, IoTST;
- An experimental evaluation of distinct TLS suites on a low-cost COTS device using IoTST.

The remainder of this paper is organized as follows: Section 2 presents the related work; Section 3 presents the main observations that led to the design and development of IoTST, its technical challenges and the proposed solution; Section 4 presents the experiments we aimed to benchmark, the metrics collected by IoTST and a comparison with other related studies; Section 5 contains the conclusions of this work.

## 2. Related Work

The Internet of Things (IoT) envisions the connection of billions of devices on the Internet, generating trillions of gigabytes of data [16]. This presents challenges in various areas of study. This section presents a literature review of how benchmarks are currently addressed in the context of IoT and its challenges, with a focus on elements relevant to the specific objectives of this paper.

### 2.1. The Need for a Proper Benchmarking Method

Edge computing addresses some IoT challenges in moving computation from cloud to the edge of the network [17]. Data-volume reduction is obtained by filtering and aggregating performed at intermediary nodes. Offloading some computing tasks from sensors to intermediary nodes also provides energy efficiency and reduced response times. Measuring edge performance started to gain attention over the last years in the form of benchmark tools. Ref. [8] performed a comparison of the characteristics of a system in existing edge benchmarks over 18 suites. Although the network plays a key role in performance, only two benchmarks address this aspect. The first aims to evaluate the overhead introduced by virtualization performed on edge servers. The second measures communication latency from a device to the Amazon cloud. The authors pointed out that additional benchmark research is required to (i) quantify the performances of accelerators and networks; (ii) identify limitations in current benchmarking approaches on real and resource-rich test beds (those that have the hardware and software capabilities to support a network protocol suite [18]); and (iii) investigate change effects, such as updates to operating systems libraries.

In [19], the authors presented the current state of art of the functional pillars of IoT and its emerging applications to motivate academicians and researches to develop real-time, energy-efficient, scalable, reliable and secure IoT applications. Highlights of the IoT system-level issues to develop more advanced real-time IoT applications are discussed. They indicated in their abstract that millions of devices exchange information using different communication standards and that the interoperability between them is a significant issue. In this context, they pointed out that: (i) the study of IoT application-layer protocols in different environments (resource-aware and resource-constrained) with different loads and diversity of data is needed; (ii) developing a strong and lightweight authentication mechanism is still challenging; and (iii) testing and validating the performances of these algorithms by incorporating them into the IoT application-layer protocols are also major research concerns.

Another challenge is the power required by these billions of devices [20,21]. Many applications follow a pattern: (i) data are acquired and (ii) processed, and (iii) information is sent through a wireless channel. This process repeats, and its duty cycle is fundamental: the smaller it is (shrinking can be achieved by shortening the active time or by lengthening idle periods), the lower the average power required [11]. As better efficiency is expected to be obtained by initiatives such as the use of edge computing, this paper moves towards the sensor nodes pursuing processing efficiency on mandatory local computation performed at every IoT sensor. Specifically, our interest is in proposing a benchmark that, in a real usage scenario, produces comparable results to local processing, considering the issues related to all three steps of the aforementioned pattern.

### 2.2. Wireless Network-State Interference

Wireless is the main channel type in use by IoT nodes. The interference that this kind of channel is subject to is also of interest. Here we indicate some work related to this subject so we can proceed in presenting IoTST’s context.

In [14], the authors studied the effect of interference to propose a multi-hop multi-channel topology control protocol for wireless sensor networks that takes into account interference caused by Wi-Fi networks in operation in the vicinity. They observed that most of the proposed protocols do not perform as per their designs when subjected to real radio environments. To illustrate this interference, they conducted experiments with eight pairs of nodes, with each pair tuned to a separate frequency. Transmitters were placed in one line at a distance of 1.5m from their respective receivers. For nodes that were also under the influence of Wi-Fi interference, they found that the average packet reception rate was above 75%. They showed that one of the major causes of under-performance is the interference issues resulting in packet drops, retransmissions, link instability and inconsistent protocol behavior.

Knowledge extraction from large-scale wireless networks is studied in [13]. It presents and characterizes neighborhood inference, and also some considerations to show how the environment in which wireless networks are immersed changes constantly. It discusses the equipment involved in wireless communication and indicates that the access points that build the network infrastructure are usually fixed and connected to the wired network structure. Nevertheless, user mobility causes fluctuation in the noise level to which the access points are subjected and interferes with the radio environment, as people’s bodies act as a barrier to microwave propagation. It also states that in large-scale networks, new access points may be switched on and off at any time; that some access points are mobile due to the proliferation of cellular Internet via Wi-Fi sharing; and that the radio environment may constantly change, for example, by opening and closing doors.

During this paper, we will indistinctly use the term *network-state interference* to refer to the effects that the constant changes in the wireless channel cause on the measurement results. This includes changes to the estimated delay, packet losses and retransmissions that might occur.

### 2.3. Current Benchmark Approaches

Here we present benchmark strategies commonly referenced in the literature so that we can discuss them when we present our method.

Real-time operating systems (RTOS) emerged as a candidate operating system (OS) to provide support to IoT devices [22]. In such systems, preemptive scheduling typically responds to events or interrupts within a required time. The authors of [23,24] presented a set of fine-grained metrics to compare RTOS distributions. The proposed metrics were based on common OS operations such as intertask synchronization and resource sharing operations. In [25], a set of benchmark tests on the selected open-source and proprietary RTOSs focused on the IoT is presented. The benchmarks are the task-switching time, the time for getting and releasing a semaphore, the time for passing a semaphore, the time to pass and receive a message, the time to pass a message between tasks, the time to acquire and release a fixed-size memory region, the time to activate a task from within an interrupt service routine and the task activation jitter. For future works, they indicate that a performance study of the network communication and routing protocols provided by the studied RTOSs, such as 6LoWPAN, RPL and CoAP, could give more insights into which OSs are best suited for various kinds of applications.

Another common benchmark approach used in the IoT context is based on the usage of commonly accepted benchmarks. The authors of [26] used such a strategy to benchmark an ESP32 device on executing the Linpack [9] and the Dhrystone [10] packages. The authors of [27] also compared their results to those obtained by the use of Dhrystone [10].

When the above approaches do not suit well the specific applications or protocols being proposed, another method commonly employed is the computing of the stop–start interval to evaluate the processing required. We present a set of related studies that use this approach in Section 4.4.

## 3. Proposed Solution: IoTST

Here we present the motivation, the technical challenges, the proposed solution and the metrics computed by IoTST.

### 3.1. Motivations

From the fact that communication is mandatory and benchmarks are not readily available, the following general observations were considered when developing IoTST:

- Metrics must detail the processing: Poorly considered idle loops can double the system’s energy requirements [28]. There are situations where industrial real-time operating systems (RTOS) miss deadlines with predictable regularity and with probability 1, even when the systems are under light loads [28]. Many benchmarked results are based on stop–start intervals, reporting only the total time spent on a specific workload. Hence, they do not provide insights on the resource usage. Examples of optimizations based on detailed resource usage are: turning off a processor in cases of much idle usage, using idle time to execute other task in parallel and choosing a scheduler quantum (preemption interval) best fitted for the tested application.
- Comparison of different standardization efforts in support of the IoT: New communication protocols, service discover protocols and operating systems running on distinct hardware [29,30] are being developed and should be compared using a common methodology.
- Agnostic-network-layer results: Once a data communication channel is chosen, we should be able to compare the processing required by different configurations. Benchmark results should be equivalent to the ones obtained in an ideal, instant and error-free channel.
- Instrumentation: A detailed benchmark implementation is a time-consuming and error-prone task, as it requires the analysis and modification of the original code and its future updates. Users should have access to the benchmark functionalities without the need for internal source code inspections or modifications. A benchmark tool should also facilitate the experimental coding.
- Overhead: A detailed information of the execution could demand extra processing, a large amount of memory or a persistence mechanism. A benchmark should quantify the processing introduced and not disrupt the usual processing.

### 3.2. Technical Challenges

IoTST benchmark results bases are scheduler traces collected, at the kernel level, from an RTOS running on a multi-processor device. An experimental result consists of multiple repetitions of code and the analysis of the produced traces. This paper investigates the production of agnostic-network-layer results using a real multiprocessor IoT device.

The main technical challenges of implementing IoTST are:

- To collect execution traces;
- To provide a classification method that relates the trace blocks to its correspondent source code blocks and to specific OS operations under analysis;
- To produce agnostic-network-layer results;
- To minimize and quantify the introduced overhead.

#### 3.2.1. Execution Traces

The production of traces containing detailed information of each task executed on each processor introduces extra processing related to the scheduler’s routine. Instrumentation code containing user-level functions such as memory allocation, inter process communication or task synchronization have large footprints and should not be used. The trace should be kept in memory, but if persistence is needed, the extra I/O processing should be identified. To produce the traces with minimal overhead and to collect every piece of scheduled task information, IoTST is implemented at the kernel level. The low complexity of kernel functions is a primary factor for the OS’s efficiency [31]. An important prerequisite for guaranteed runtimes of O(1) is the exclusive use of static memory allocation in the kernel. IoTST code should be optimized, as it will execute on every call to the scheduler routine. It should also provide a method so that we can associate trace records with the experiment source code, as we present next.

#### 3.2.2. Classification

Within the trace, we want to identify the occurrences of specific events. Classification associates a group name to future trace records. The final user uses this functionality to associate trace records with the experiment source code. Internally, IoTST use it to identify records created after the execution of code of interest. The challenge here is to guarantee the correct distribution of the current running tasks’ statistics between the previous and the newly created group. When the Classification function is called, it should compute the statistics of each task currently running on each processor and classify it as part of the previous existent group. This requires the deviation of each processor from its task to a specific routine and synchronized resuming of each task after the new group’s creation. In addition, we have to compute the overhead introduced, especially the inter processor’s sync waiting time.

#### 3.2.3. Agnostic-Network-Layer Results

**Figure 1:**
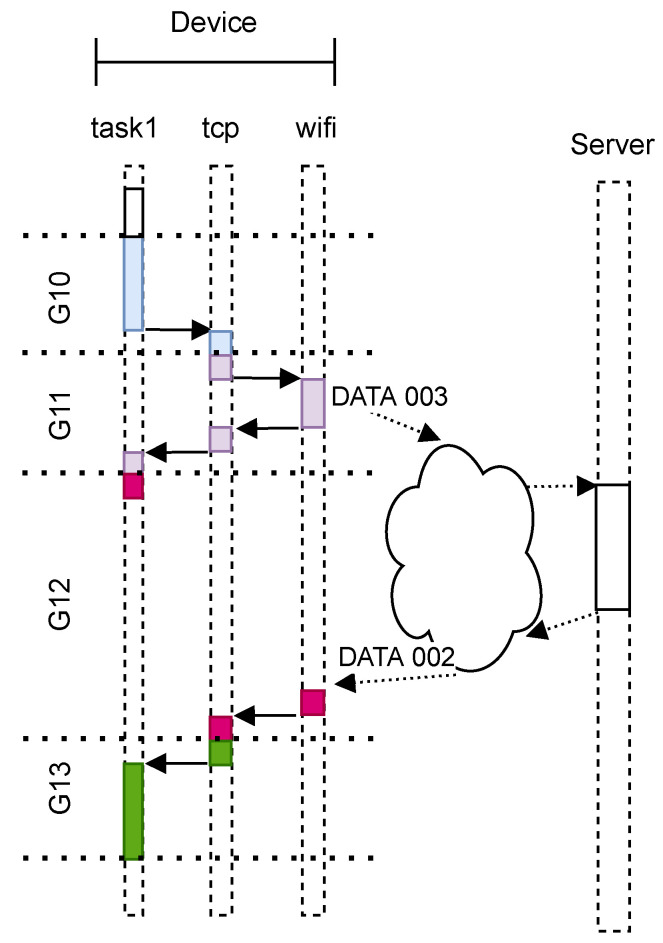
Local task grouping example when benchmarking communication.

To produce agnostic-network-layer processing analysis, we need to identify when the device is idle and wait for data transmission to complete. We must also discard an experimental repetition when retransmission occurs. Classification identifies when internal OS network events occur (i.e., connection on data transmission). In this paper, we use the TCP/IP protocol. Network traffic is classified by inspecting every package sent or received. We verify if it contains a special flag, i.e., SYN, or application data. For each verified case, we have a counter. A new group is created on every package inspection and named according to its type and counter value (i.e., *SEND_SYN_000*, *RECEIVE_DATA_001* or *SEND_DATA_002*). The Inspection should be lightweight; otherwise, we could cause a retransmission if TCP timers exceeds. We only check if a retransmission occurred at the end of the current run, when the recording phase is off. Figure 1 shows tasks interaction and grouping when communication occurs.

### 3.3. Proposed Solution

**Figure 2:**
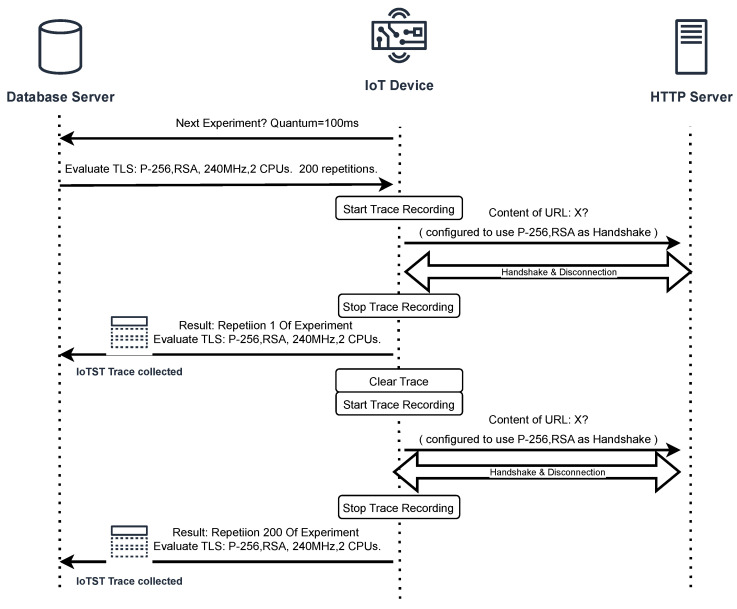
Generic IoTST computing architecture.

To verify the implementation and execution of IoTST, we developed a complete environment to support the orchestration of the evaluations being performed. Figure 2 shows an overview of IoTST requesting the experiments to be executed and reporting each repetition’s trace results. In the following sections, we will describe the details of its implementation.

#### 3.3.1. Benchmark Based on Scheduler Traces

**Figure 3:**
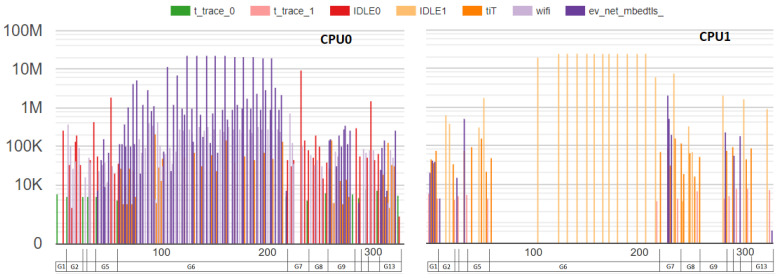
Scheduler trace example. A device with 2 CPUs running at 240Mhz. On the vertical axis, on the logarithmic scale, is the number of cycles used by each task when occupying a CPU. The horizontal axis presents the step in which the scheduler removes the task from execution, in reference to a single scheduling counter of the experiment. Task names are presented along the top.

As we present in Section 4, many benchmark results are collected using a stop–start interval and present only the overall execution time. IoTST’s approach is based on the collect and classification of detailed execution traces, at the kernel level, as the one presented in Figure 3. From trace analysis, IoTST provides the three metrics presented in the next subsection. The analysis of IoTST traces assists in the evaluation of possible configurations, code optimizations and the determination of the optimal operating point. This kind of analysis is not possible when considering only the total execution time as a benchmark.

#### 3.3.2. Classification

Classification and isolation of its overhead is a key feature of IoTST. During the initialization, IoTST creates, for each processor, a pinned task called *t_trace*. Each *t_trace* has the highest system priority and stays suspended, waiting for a new group notification. On a group creation call, all *t_traces* are notified to be ready to execute. The running tasks leave their processors, and the trace record is associated with the existent group. Any *t_trace*, once started, occupies its processor until all *t_traces* also occupy their respective ones. A new group is created, and all new records will be associated with it. Quantification of the introduced overhead, such as the group creation or the need for partial persistence of large execution traces, is associated with a *t_trace* record, and optionally discarded, when computing the benchmark. Using classification, metrics are not limited to the overall execution; they can be locally analyzed, in relation to distinct source code blocks and the executed tasks. Classification also complements the trace with internal OS information. Users of the IoTST can have their traces grouped by the occurrence of specific internal OS events, negating the need to inspect the internal OS’s code. In the works presented in Section 4, this kind of analysis is not supported, so one cannot, for example, investigate local possible optimizations. In addition, the works presented in Section 4 do not address the verification of the introduced overhead.

#### 3.3.3. Agnostic-Network-Layer Results

This is the main problem that IoTST aims to address in this paper. When an experiment uses a stop–start interval to evaluate code that involves network traffic, the results can be influenced by network-state interference. To allow the comparison of different standardization efforts in support of the IoT, IoTST proposes that comparable benchmark results should be produced considering an ideal and error-free communication channel.

To address this problem and to point out that many works neglect the network-state interference in their results, IoTST is described, and it was implemented and used to benchmark an experiment that involves network communication. It was used to evaluate the results and their respective confidence levels in two cases: the case where network-state interference is neglected and the case where benchmark results are obtained using the proposed IoTST method. This is not the case for the methods presented in Section 4 which involve network transmissions. They do not consider how their results are influenced by their current network state.

#### 3.3.4. Use and Portability

It is important for a network’s downstream applications to have IoTST implemented in an efficient way and that the user can use it as a plug-and-play module.

IoTST is implemented at the kernel level, exposing network-related events through the trace collected. The processing overhead introduced by the *t_trace_0* task when a new classification is performed is fully evaluated through trace collection. The *t_trace* records are not taken into account when the final results are computed by the report/database server, ensuring the accountability and accurate measurement of the introduced overhead, without affecting the validity of the benchmark results.

To orchestrate and facilitate coding, the macros presented in Listing 1 are provided by IoTST. One-time executed code, such as declarations, allocations and initializations, should be in the macro *EVLTR_pre*. The processing related to this code block will not account for the results of each run. The code to be evaluated should be placed in the macro *EVLTR_run*. This block will repeat as many times as requested. Internally, the *EVLTR_run* macro uses a surrounding loop instead of performing additional function calls, thereby avoiding the introduction of extra code related to deviation operations, such as register, save and restore. The trace recording only starts after *EVLTR_run* creates its first group and automatically finishes after each repetition. Each code block inside this macro can create as many groups as needed so that the results can be classified during the analysis. We use an error flag to optionally discard the collected trace if we can predict that an error occurred (i.e., could not allocate memory or did not receive an answer from the server). After each successful repetition, the recording phase stops, and its trace is sent to a server.
**Listing 1.** Proposed macro for an experiment evaluation.
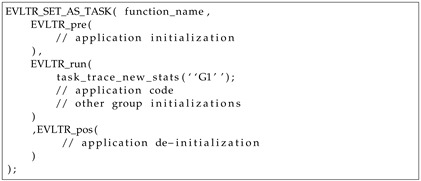



As a kernel modification, IoTST uses low-complexity functions and static memory allocation. It can be distributed as a compiled version of the OS or as a patch. To code using its current functionalities, the final user is only required to code experiments, introduce the desired classifications throughout the code and setup its environment, as described in Section 5.

The port of IoTST to other RTOS or testbeds, considering the technical challenges presented, is possible, but will be investigated in another publication.

### 3.4. Metrics Computed by IoTST

#### 3.4.1. Cycles Used

As an experiment, we collected the trace of each run. Each trace is an ordered list of each task executed by the scheduler. Each element of the list contains: a task name, a group name, the number of cycles used in that run, the CPU used and the number of cycles used by the scheduler to select the task for execution. We did not investigate scheduler’s performance here and considered the sum of both counters as the element’s cycle counter (ecc). We computed each trace’s total cycles (ttc) as the sum of its element’s cycle counter (ecc). An experimental benchmark is the average of its ttc values. The margins of error were calculated based on a 95% confidence interval.

**Figure 4:**
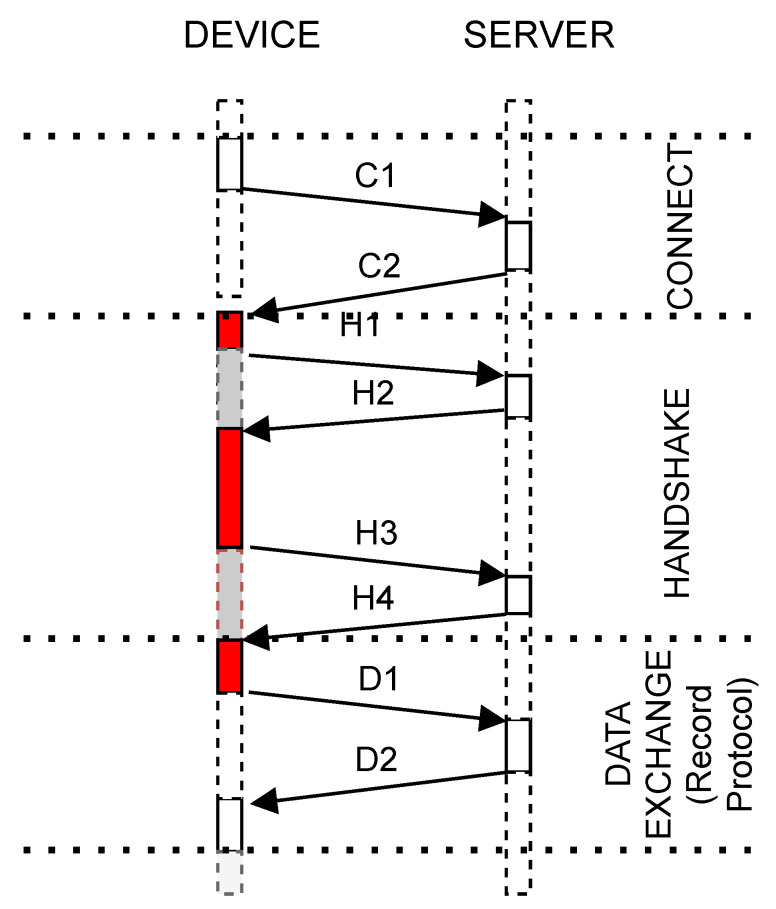
Examples of messages between a device and a server when using TLS.

In order to investigate network-state interference, we used two criteria. For the first, we discarded from the traces the elements belonging to the groups associated with the time in which the device was waiting for the sending or receiving of network data. The experiment repeated until we had enough traces to produce an error level of above 1%. This result is considered network-invariant. In Figure 4, this is the case where only the red groups are considered. For the second criterion, we observed how the benchmark value changes when the same collected traces are considered with no group discarding. This time, we observed not only the benchmark value, but also its error. This is the case where both the red and the gray groups from Figure 4 are considered.

#### 3.4.2. Minimal Supported Quantum Slice

An inherent characteristic of real-time systems is that their requirements include time information in the form of deadlines [32]. They are designed to provide deterministic timing, meaning that they guarantee specific and predictable response times for different tasks.

This metric aims to investigate the OS with respect to its time quantum/time slice property to evaluate its real-time properties [30].

A task should not execute, uninterruptedly, for more than the OS’s quantum value. A preemption should occur, at most, in the specified quantum time so that the OS scheduler’s routine can execute on each CPU to check and decide on the next task to execute, according to its scheduling policies. If this property is not satisfied during a task execution, we want to be able to determine a value that satisfies this mandatory RTOS characteristic.

Concerning the four main blocks of an IoT application presented earlier, knowing the quantum value is relevant to the determination of the minimum interval supported between executions of sensing or actuating functions, i.e, minimum supported sampling time.

#### 3.4.3. CPU Usage

This metric quantifies unused cycles per CPU—idle time. This aims to investigate if a configuration where CPUs are turned off provides satisfactory performance, or whether other tasks can be introduced to run in parallel.

## 4. Experiment Results and Comparison

To demonstrate IoTST, the protocol that we benchmarked in this paper was Transport Layer Security (TLS) [33]. The experiments aimed to benchmark, on a real COTS device, the handshake phase of the TLS protocol using NIST’s minimum recommended security level to be adopted from the year 2030 on, which will be 128 bits [34]. In its last version, TLS 1.3 uses elliptic curves (ECC) [33] for the key exchange phase, known as ECDHE. For the authentication phase, ECC is known as the ECDSA [35], and RSA certificates are supported. We consider the case where the device connects to a server as a client and exchanges the necessary messages, and the client receives a certificate and verifies the server’s identity.

The following TLS and device configurations were benchmarked: Two key exchange-phase curves, Curve25519 [36] and P-256 [37]; two authentication-phase algorithms, the RSA and the ECDSA with P-256; and two CPU modes, single or dual activated CPUs. We obtained metrics for distinct operating frequencies, as it can be configured in most platforms, and presumably could have a high impact on the performance of algorithms or existing hardware accelerators. We also investigated the minimal quantum slice that a task uses while performing handshake operations.

The experiments had the following objectives: (i) to show how network-state interference affects the benchmark results, (ii) to investigate how IoTST can contribute to the production of comparable results, (iii) to present benchmark results using different hardware and software configurations and (iv) to be able to discuss the results.

First, we present a brief description of the protocol. Next, we present the implementation details, followed by the results. We conclude this section with a discussion and comparison with related works.

### 4.1. Transport Layer Security—TLS

When performing secure communication, TLS is used by many of the existent high-level protocols proposed for IoT [38,39]. Figure 4 shows an example of messages exchanged between a device and a server. A connection is established when a device sends a C1 package containing a SYN flag and receives a C2 package, also containing a SYN flag. The handshake phase is then started. The red blocks represent the groups we want to benchmark, which comprise all the handshake phases. The gray ones represent the time in which the device is waiting for the sending or receiving of messages. The first red block represents the group wherein the device is preparing the *ClientHello* package *H1*. During the second red block, the device parses the TLS messages received from the server. As described by [37], these messages are *ServerHello*, the server’s certificate message, the *ServerKeyExchange* and the *ServerHelloDone*. During the second red block is when the authentication and key exchange mechanisms’ computations are performed. A detailed description of the TLS can be found in [37,38,40,41].

### 4.2. Implementation Details

#### 4.2.1. Selected IoT Device

**Figure 5:**
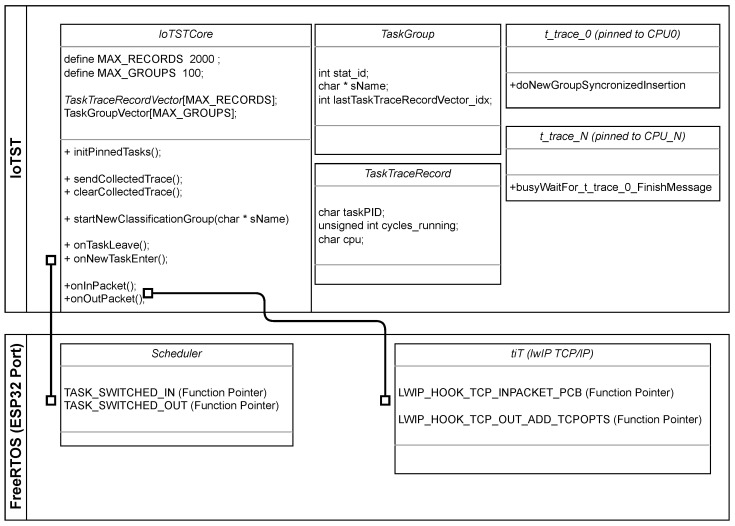
Relevant C code’s organization. The lines between IoTST and the RTOS packages indicate the functions linked during compilation.

We conducted the experiments on a COTS IoT device, ESP32 [42]. This is the same one selected by most of the papers presented in the previous section. ESP32 OS is based on the open-source FreeRTOS [43]. The firmware version used was 4.1 [44]. Figure 5 depicts IoTST’s main functions and how its linked to FreeRTOS at the kernel level during compilation.

For the authentication and key exchange phases, the firmware uses the ECC and RSA, which were proposed by TLS 1.3. ESP32 is a SoC that has a 32-bit LX6 dual-core microprocessor and an IEEE 802.11b/g/n interface. Each CPU can operate at 80, 160 or 240 MHz. The hardware acceleration engine supports the AES, SHA-2, RSA and ECC cryptography algorithms. The power consumption, on a 802.11n network while the radio is active, is stated to be the same for any operating frequency [42]: 180 mA when transmitting with a 50% duty cycle, or between 95 and 100 mA while receiving. The device used a 3.3 V regulated supply as its power source. The default network stack, lwIP [44], was used with its TCP’s default maximum segment size (MSS) of 1440 bytes.

On an ESP32, the described IoTST methodology makes use of specific tasks, OS macros and firmware libraries, as described:

- Task scheduling: When a task is scheduled, the macros TASK_SWITCHED_IN() and TASK_SWITCHED_OUT() [43] are called. We use them to collect the trace.
- Idle time: A CPU in idle state executes a specific pinned task (IDLE0 for CPU0 or IDLE1 for CPU1). When analyzing the trace, we compute the idle time by the investigation of the cycles used by these tasks.
- Network communication inspection: The tiT task executes the TCP-related functions, and the wifi task executes the MAC/PHY-related ones. The manufacturer does not provide the source code for the network driver. To inspect network communication, we used the lwIP TCP library macros LWIP_HOOK_TCP_INPACKET_PCB LWIP_HOOK_TCP_OUT_ADD_TCPOPTS.

**Figure 6:**
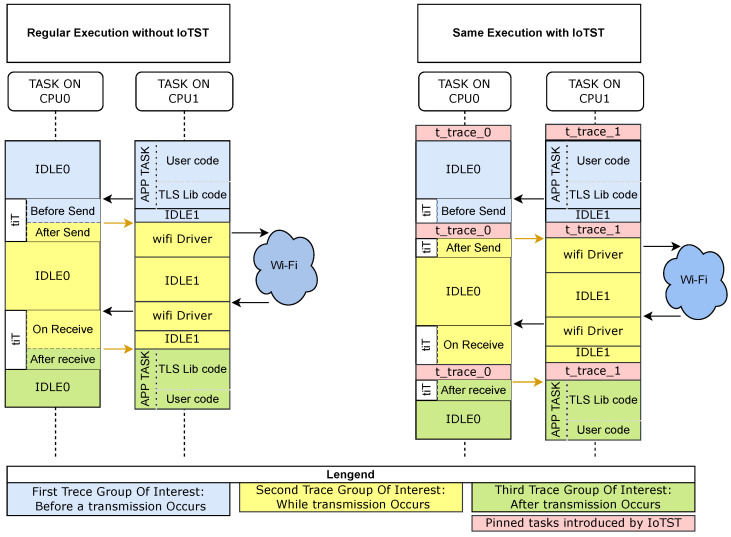
Regular execution and the same execution with the use of IoTST.

Figure 6, on the left, demonstrates a regular execution. If, for example, a start–stop timer is introduced before and after the original execution, it will not give any information about the parallel execution performed. That is not the case when the traces collected by IoTST are analyzed.

#### 4.2.2. Certificate Generation and Hosting

A server running CentOS 7 [45] created the signed certificates. The certificate and its signature were of the same type (RSA or ECDSA using the P-256 ECC curve). The authority’s public keys were uploaded to the device so that it can verify the signatures. Thus, four different urls were provided by the web server, each one using a distinct combination of the two key exchange algorithms and the two certificates.

#### 4.2.3. Data Collection

We compiled the experiments and uploaded them to the device. Once booted, it sends a request to the server. The server responds with the next experiment to perform, the number of repetitions to execute, the URL to connect to and the frequency that the device should use. The device sets its frequency and enters in a loop. After each repetition, a trace is sent to the server. As presented, each experiment was coded using the macros presented in Listing 1 so that its trace-recording phase, on each repetition, would start or stop accordingly. When all the successful repetitions are completed, the device requests another experiment to run. The server uses a data base management system (DBMS) to save the trace, to check the next experiment to be executed, and then calculates the benchmark.

### 4.3. Results

The results obtained are presented as: (i) accuracy verification, (ii) CPU usage, (iii) handshake analysis and (iv) quantum analysis.

#### 4.3.1. Accuracy Verification

This subsection numerically presents how network-state interference affects the results. Table 1 shows the results with no discard while Table 2 shows the results obtained with the discard of the groups associated with data-transmission waiting.

In order to obtain a margin of error above 1% using a 95% confidence interval, a total of 4800 traces were needed—200 repetitions for each possible key exchange, authentication, frequency CPU configuration. On average, each trace collected had 177, 352 and 467 elements for the frequencies of 240, 160 and 80 MHz, respectively.

A total of 1,201,937 task info elements were collected. For the same 200 repetitions using Curve25519 and one CPU, Table 1 shows the results when the groups associated with data-transmission waiting are not discarded. For the 240 MHz case, there is an overlap of the 95% confidence interval for the RSA (0.68 s, 1.50 s) and the ECDSA (1.42 s, 2.14 s), indicating that the difference between the experiments may not be significant.

By comparing Table 1’s results with the respective results in Table 2, we can check network-state interference’s effects on the obtained confidence. On discarding the groups associated with data-transmission waiting, we are 95% confident that the mean of the Curve25519 at 240 MHz was between 0.577 and 0.582 s when using the RSA and between 1.393 and 1.407 s when using the ECDSA. This illustrates one of IoTST’s goals being achieved: the need for a proper benchmark, capable of producing comparable and high-confidence results, when the processing of applications that communicate over the network are to be benchmarked.

#### 4.3.2. CPU Usage

Table 2 shows that the single-core experiments outperformed the two-cores ones when all other parameters were the same. We also calculated the CPU occupancy using the collected traces. For the single-CPU case, the IDLE task was executed for less than 1% of the total time. This indicates that the CPU was almost totally used when the handshake was not waiting for data. When using two CPUs, the IDLE tasks was executing 47% to 48% of the time. This configuration is only justified if another task is running in parallel, while the handshake is performed. A handshake implementation that performs parallel calculations [46] might also improve the CPU usage in this scenario. In [47], the authors presented the challenges on implementing parallelism to the Crypto-Subsystem and highlighted that the currently available IoT OSs are not optimized for parallel processing because real-world IoT firmware is tailored to a single application. In this scenario, it is up to the application developer to compare their CPU usage to exploit parallelism in order to obtain shorter periods of inactivity and the consequent decrease in the average energy required [11].

#### 4.3.3. Handshake Analysis

Table 2 shows that Curve25519 outperformed P-256 when the other settings were the same. At 240 MHz, the selection of Curve25519 and RSA can roughly provide a 4× speedup over the P-256 and ECDSA configuration. If the ECDSA is selected, using Curve25519 at 240 Mhz can roughly provide a 2× speedup over the P-256. These speedups consider an ideal, instant, error-free channel and require some observations.

On considering the exchanged TLS messages, the key size of each authentication algorithm will introduce distinct delays. As the RSA’s key size is 3072 bits and the ECDSA’s is 256 bits, on an ideal 300 Mbps wireless channel, the key transmission of the RSA takes 10.2 ms, whereas the ECDSA takes 0.8 ms. Although this delay is not significant regarding the mean time presented, it might not be the case on a degraded channel.

In order to proceed in our analysis, we will consider, based on TLS 1.3 specifications [33], that when a full handshake is performed, each TLS 1.3 message type, except the one containing the certificate, has roughly the same size regardless of the crypto suite selected. With this assumption, we consider that if the same channel state is given and the same device configuration is used, the device transmission time of the exchanged messages would be the same regardless of the suite used.

While the device is receiving the server certificate, we can use Table 2 to estimate the channel speed so that the RSA would take the same length of time as the ECDSA, when all other configurations are the same. For example, at 240 MHz using one CPU and Curve25519, the difference between RSA and ECDSA configurations is 0.82 s. Thus, to have the same processing and certificate receiving time, a channel speed of (3072−256)/0.82 s = 3434 bit/s is required. At this speed, both take 1.47 s plus the same certificate transmission time contribution. The ECDSA would outperform the RSA only if the channel speed is below 3434 bits/s. With the same considerations, we can estimate the energy consumption difference. In the above example, if a perfect channel is considered, the ECDSA would require at least an additional (3.3 V × 100 mA × 0.82 s) = 270 mJ when compared to the RSA.

#### 4.3.4. Quantum Analysis

Quantum analysis is performed by the inspection of the per processor traces collected and the identification of the maximum run time of every task selected by the scheduler. The system under test’s default quantum value is 10 ms. If any task executes for more time than the specified quantum, the system is not satisfying its real-time properties, as we discussed in the previous sections.

Using the traces collected from the previous experiments, we checked for any task that occupied the CPU, uninterruptedly, for more time than the default quantum. As each line of each trace contains this information, we generated a report containing, per experimentation configuration (crypto suite, number of CPUs and clock), the total number of occurrences per task, per classification group.

By analyzing the collected traces from previous experiments using a database (Figure 2), we identify any task that continuously uses the CPU for a longer period than the default quantum. The information on each task’s CPU usage is contained in each trace line, which we use to generate a report by filtering these records. The report lists the task name, the number of occurrences that exceeded the quantum time, the IoTST classification group and the experimentation configuration (cryptographic suite, number of CPUs and clock speed).

Our analysis of the report showed that during the handshake process, the system exceeded the maximum quantum time and executed the same task for more than 95 ms and less than 100 ms, regardless of the configuration used. As the network driver is not open-source, we were unable to inspect its code, but we assume that the scheduler is deliberately suspended while the crypto accelerator is in use.

We then conducted the same experiments described in Section 4.3.1, this time with a quantum value of 100 ms. The newly generated report indicated that no task consumed the CPU for more than this duration. Based on these findings, we conclude that 100 ms is the minimum quantum value that should be used by the device during a TLS 1.3 handshake using the evaluated cryptographic suites.

### 4.4. Discussion and Comparison with Related Works

Some of the additional benchmark research needed, pointed out by [8], was addressed in this paper. To investigate change effects and to identify limitations in current benchmark approaches, we provide a method that details the processing with a known overhead. In our accuracy verification experiment, we demonstrated how the benchmark can be influenced by the network state if not considered. We then quantified the current network state’s influence and subtracted it from our results. We also subtracted the introduced overhead so that we could provide a benchmark that allows the comparison of different standardization efforts in support of the IoT.

In regard to the fine-grained metrics to compare RTOS presented in Section 2 [23,25], aside from providing a comparable method for evaluation, this approach does not provide a good indicator for an application performance, as the underlying libraries might use different combinations of these fine-grained metrics. A map of the application to the fine-grained metrics might not be trivial or could even be unfeasible if some libraries do not provide the source code, so the evaluation on a real scenario might be difficult or unfeasible.

The usage of common accepted benchmarks, such as Linpack [9] and the Dhrystone [10] packages, suits comparing devices performing the same operations, but lacks when it comes to providing specific application performance. In addition, the characterization of externally influenced operations, such as network communication, is not addressed.

As far as we know, we are the first to propose an IoT application benchmark method that standardizes the production of network-invariant comparable results. Nevertheless, many studies have presented benchmarked results. In this study, we used IoTST to evaluate TLS on an ESP32 [42] device. In the following publications, closely related to our work, existent and new protocols were proposed and evaluated in specific scenarios, using various methodologies. We proceed with a discussion to highlight how IoTST compares or contributes to the following works.

Post-quantum cryptography (PQC) algorithms were coded by [40] into the mbed TLS library, and the full TLS handshake performance was benchmarked. The ECDSA, using the curve P-256, was also benchmarked. The handshake was evaluated using three devices, including an ESP32. Performance was measured at the level of the handshake state machine. As mentioned by the authors, their measurements did not include the network-stack overhead but only the time taken by the handshake routines. They measured each test case multiple times and reported the minima of the measurements. As IoTST, their approach is not influenced by the current network state, but it does not compute the total processing required by the interaction between the involved libraries. They reported only the minimal measurements. IoTST’s confidence-interval approach would verify if the presented result was a good estimation of the expected average time, and in a real usage scenario. IoTST would also provide insight on the CPU usage so we could further investigate if any OS parameter change, for example, a different quantum value, could benefit their results.

The performances of different algorithms concerning data integrity, authenticity and confidentiality were evaluated by [41], using three devices, including an ESP32. For each experiment repetition, they computed the stop–start interval, including the wireless communication performed. They indicated that the processing associated with the public keys exchanged between the two parties was not evaluated to avoid incorrect measurements which take network latency into account, which could be affected by environmental specific factors such as signal interference. IoTST would address the network-state interference issues pointed by the authors and provide comparable results.

The authors of [48,49] used stop–start-interval-based measurements to evaluate different crypto suites on an ESP32 device. They also used a security level of 128 bits, as did our experiments. They repeated their experiments 100 times. Each experiment involved a connection, and the stop–start interval, while establishing secure communication as a client, was computed. They indicated that they could not obtain satisfactory results in some experiments due to the large delays introduced in the different phases of the communications protocols, i.e., during the TCP and TLS handshakes. They proposed software optimizations on the HTTPS server as one of the alternatives that might mitigate those effects. In their conclusions, the authors stated, with no justification, that the reason behind this degradation in performance was the communications delay. They stated that: “ECDSA presents itself as a better alternative than RSA for securing resource-constrained IoT devices”. With the same configuration parameters reported by the authors, IoTST results, with the discarding of the network state, showed that the RSA outperformed the ECDSA. To provide a 128-bit security level, the RSA requires a 3072-bit key size, whereas the ECDSA requires a key size of 256 bits. By using IoTST instead of computing the overall stop–start interval, we detected that an extra TCP packet is sent when the RSA is selected. We conclude that IoTST can contribute to: (i) allowing the comparison of network-invariant results; (ii) checking the number of exchanged packages to identify and discard experiments on the occurrence of retransmissions that might have influenced their results; and (iii) investigating if a change in an OS parameter (such as the default maximum segment size) might reduce the number of TCP package transmissions and contribute to their results.

In [50], extensive experiments were performed to validate the performance of the cryptographic and networking operations of IoT devices, including the ESP32. They also evaluated the ECDSA using a security level of 128 bits, but for the RSA, they used a security level of 80 bits. All metrics were obtained 100 times. Although they compared distinct security levels, they reported that the RSA’s variance was significant, and that the RSA’s operations fared much better than ECDSA’s due to hardware acceleration. We consider that both the ECDSA and the RSA should be compared with no network-state interference but with the same security level and that the IoTST should be used to measure the overall system’s processing performance (including all the libraries interactions involved) to provide a better estimation of a real usage scenario. For example, buffer transfers among the libraries involved, the hardware accelerator and the network-state interference can contribute to distinct conclusions on a real usage evaluation. Additionally, any race condition for resources would impact the results. We also highlight that the consideration that RSA’s operations fared much better than the ECDSA due to hardware acceleration provided by ESP32 should be investigated when both are using the same security level. If confirmed, this should also be noted by the authors of [48,49].

In [51], the authors investigated the side-effects of CoAP and MQTT protocols acting as the two end-nodes of the network, using the Arduino platform. They reported that nodes are located at a distance of 1m from each other and that the experiments occurred in standard room conditions. They computed the stop–start interval, including the wireless communication performed, and concluded that it takes about 4.99 times more time for a MQTT client to receive the contents. They stated, with no quantification, that one of the major reasons for this difference in the latency of packet delivery in MQTT and CoAP is the sliding window mechanism employed by the TCP flow control, and that another is the three-way handshaking required for initializing the communication in MQTT. For this work, we also propose the use of IoTST instead of computing the stop–start interval considering the network transmission time.

The authors of [41] investigatde blockchain usage to support distributed artificial intelligence. They used ESP32 IoT devices to participate in blockchain mining. They measured the number of hashes that a device can perform per second (h/s) and reported that ESP32 averaged 17.4 Kh/s using a not-specified configuration. The IoTST CPU benchmark can be used to investigate if parallel hash calculations could improve the overall performance. IoTST quantum analysis could help in the investigation of a new quantum value best-fitted for this application so that less processing is dedicated to unnecessary scheduling attempts when no other task is selected.

## 5. Conclusions

Currently, there is no available IoT benchmarks that addresses the following question: how can one quantify and compare the relative performances of IoT applications or protocols that communicate over a wireless channel, while disregarding the influence of changes in the state of the network?

We discussed how some of the addressed papers use a different methodology and sometimes make use of not-quantified assumptions when trying to circumvent the problems that network-state interference may cause to their results.

We addressed the above question and presented IoTST, a benchmarking suite based on per-processor synchronized stack traces with the isolation and precise determination of the introduced overhead. We implemented IoTST at the kernel level. As a benchmark approach at the kernel level, it offers several benefits for the evaluation and optimization of IoT devices. One of the key advantages of this approach is the low overhead it introduces, allowing for accurate and precise measurement of system performance. Additionally, the kernel level provides access to specific data collection that cannot be obtained at higher levels of the system, such as the utilization of CPU resources and the behavior of system calls. This level of detail is particularly useful in a multiple-CPU RTOS environment, where the interactions between different CPU cores and the operating system can have a significant impact on device performance. By benchmarking at the kernel level, developers and researchers can gain a deeper understanding of the behavior of IoT devices, enabling them to make more informed decisions about performance optimization and design.

We have demonstrated the feasibility of using IoTST by implementing it on a real device and utilizing it to assess the impact of network state on confidence error margins. Our results reveal that IoTST classification and the elimination of groups related to network transmission result in highly confident and comparable outcomes. We compared different TLS handshake crypto suites using IoTST, which effectively measured unused CPU resources, determined the minimum quantum value required for the OS to operate and provided consistent results for benchmarking IoT applications or protocols that communicate via a wireless channel. IoTST establishes some of the necessary rules and metrics for comparison to accurately quantify and compare IoT protocols.

In this research, our primary aim was to present the IoTST methodology and evaluate its practicality on a real device. The task of coding at the kernel level is a complex and demanding challenge, requiring a deep understanding of the workings of operating systems and their internal mechanisms. One of the major obstacles in this endeavor is the lack of transparency in the internal implementations of operating systems, particularly due to the restriction of access to some critical portions of code by manufacturers for strategic reasons.

In future work, we intend to expand our research by applying IoTST on a diverse range of IoT devices and providing a comprehensive comparison of the results. Efforts will also be made to identify and create new metrics that can aid in the selection of the best crypto suite for a given network state. For instance, a metric that quantifies the current network speed will be developed. Additionally, we plan to re-evaluate our results using the newly developed FreeRTOS-Plus-TCP instead of the current lwIP library used by the ESP32 device.

## Figures and Tables

**Table 1:** Accuracy when network delay is considered. Confidence interval above 16%.

			1 CPU			
**Key Exchange**	**Auth.**	**MHz**	**Mean Cycles (/10)**	**95% CI**	**Mean Time (s)**	σ
Curve25519	ECDSA	80	387,520	±23.37%	4.84	5.97
		160	391,974	±29.87%	2.45	3.18
		240	427,420	±20.45%	1.78	2.14
	RSA	80	187,631	±16.73%	2.35	2.74
		160	216,024	±47.54%	1.35	1.99
		240	260,786	±37.85%	1.09	1.50

**Table 2:** Handshake performance when network delay is discarded.

			1 CPU			2 CPUS		
**Key Exchange**	**Auth.**	**MHz**	**95% CI**	**Mean Time (s)**	σ	**95% CI**	**Mean Time (s)**	σ
Curve25519	ECDSA	80	±0.37%	4.20	0.11	±0.50%	5.03	0.18
		160	±0.33%	2.08	0.05	±0.56%	2.55	0.10
		240	±0.29%	1.40	0.03	±0.48%	1.70	0.06
	RSA	80	±0.56%	1.76	0.07	±0.60%	1.99	0.09
		160	±0.48%	0.87	0.03	±0.54%	1.02	0.04
		240	±0.48%	0.58	0.02	±0.53%	0.68	0.03
P-256	ECDSA	80	±0.38%	4.96	0.14	±0.41%	6.05	0.18
		160	±0.47%	2.99	0.10	±0.33%	3.53	0.08
		240	±0.42%	2.27	0.07	±0.32%	2.74	0.06
	RSA	80	±0.54%	2.04	0.08	±0.53%	2.49	0.10
		160	±0.43%	1.30	0.04	±0.42%	1.52	0.05
		240	±0.52%	1.01	0.04	±0.45%	1.19	0.04

## Data Availability

Not applicable.
